# The Effects of Seasonal Variation on the Outcomes of Patients Undergoing Off-Pump Coronary Artery Bypass Grafting

**DOI:** 10.31083/j.rcm2512456

**Published:** 2024-12-24

**Authors:** Ling Wu, Pei-shuang Lin, Yun-tai Yao, on behalf of the Evidence in Cardiovascular Anesthesia (EICA) Group

**Affiliations:** ^1^Department of Anesthesiology, The First Affiliated Hospital of Anhui Medical University, 230022 Hefei, Anhui, China; ^2^Department of Anesthesiology, Fuwai Hospital, National Center for Cardiovascular Diseases, Peking Union Medical College and Chinese Academy of Medical Sciences, 100037 Beijing, China; ^3^Department of Cardiovascular Surgery, Quanzhou First Hospital Affiliated to Fujian Medical University, 362000 Quanzhou, Fujian, China

**Keywords:** seasonal variation, off-pump, coronary artery bypass grafting, complications, outcomes

## Abstract

**Background:**

The impact of seasonal patterns on the mortality and morbidity of surgical patients with cardiovascular diseases has gained increasing attention in recent years. However, whether this seasonal variation extends to cardiovascular surgery outcomes remains unknown. This study sought to evaluate the effects of seasonal variation on the short-term outcomes of patients undergoing off-pump coronary artery bypass grafting (OPCABG).

**Methods:**

This study identified all patients undergoing elective OPCABG at a single cardiovascular center between January 2020 and December 2020. Patients were divided into four groups according to the season of their surgery. The primary outcome was the composite incidence of mortality and morbidity during hospitalization. Secondary outcomes included chest tube drainage (CTD) within 24 h, total CTD, chest drainage duration, mechanical ventilation duration, and postoperative length of stay (LOS) in the intensive care unit (ICU) and hospital.

**Results:**

Winter and spring surgeries were associated with higher composite incidence of mortality and morbidities (26.8% and 18.0%) compared to summer (15.7%) and autumn (11.1%) surgeries (*p* < 0.05). Spring surgery had the highest median CTD within 24 hours after surgery (640 mL), whereas it also exhibited the lowest total CTD (730 mL) (*p* < 0.05). Chest drainage duration was longer in spring and summer than in autumn and winter (*p* < 0.05). While no significant differences were observed in mechanical ventilation duration and hospital stay among the four seasons, the LOS in the ICU was longer in summer than in autumn (88 h *vs.* 51 h, *p* < 0.05).

**Conclusions:**

The OPCABG outcomes might exhibit seasonal patterns in patients with coronary heart disease.

## 1. Introduction

The impact of seasonal patterns on the mortality and morbidity of surgical patients with cardiovascular diseases has gained increasing attention in recent years, shedding light on the complex interplay between external environmental factors and cardiovascular health [1, 2, 3]. Several contributing factors, including low temperature, respiratory infections [4], and seasonal mood changes [5], have been implicated in influencing the incidence rate and mortality of coronary heart disease. Notably, there is a discernible peak in winter and a trough in summer, suggesting a potential connection between seasonal variations and cardiovascular outcomes.

Whether seasonal variation extends to coronary artery bypass graft (CABG) surgery outcomes has been a subject of ongoing debate. While some studies have identified seasonal fluctuations, a significant degree of inconsistency persists among these findings. For instance, a comprehensive study in the United Kingdom analyzed data from 12,221 patients who underwent cardiac surgery over ten years, revealing higher risk-adjusted hospital mortality and prolonged length of stay (LOS) during the winter months [6]. Similarly, a study by Torabipour *et al*. [7] in Oman found that the surgery season significantly affected the length of hospital stay after CABG, with winter showing the longest duration and summer the shortest.

Contrastingly, other research has failed to establish significant seasonal differences in surgical outcomes for CABG patients. A retrospective cohort study conducted in Turkey, comparing winter and summer surgical outcomes, concluded that despite the increased frequency of cardiovascular events in winter, early surgical outcomes of CABG remained unaffected by seasonal patterns [8]. Another study in Iran by Nemati [9] found no significant differences in postoperative mortality, morbidity, or LOS among the four seasons.

However, it is crucial to acknowledge the potential geographical variability of these findings, preventing the derivation of generalized conclusions. Consequently, the current study aims to contribute to this discourse by assessing the correlation between seasonal variations and the outcomes of off-pump coronary artery bypass graft (OPCABG) surgery.

## 2. Materials and Methods

### 2.1 Study Design and Patient Population

**Fig. 1.**
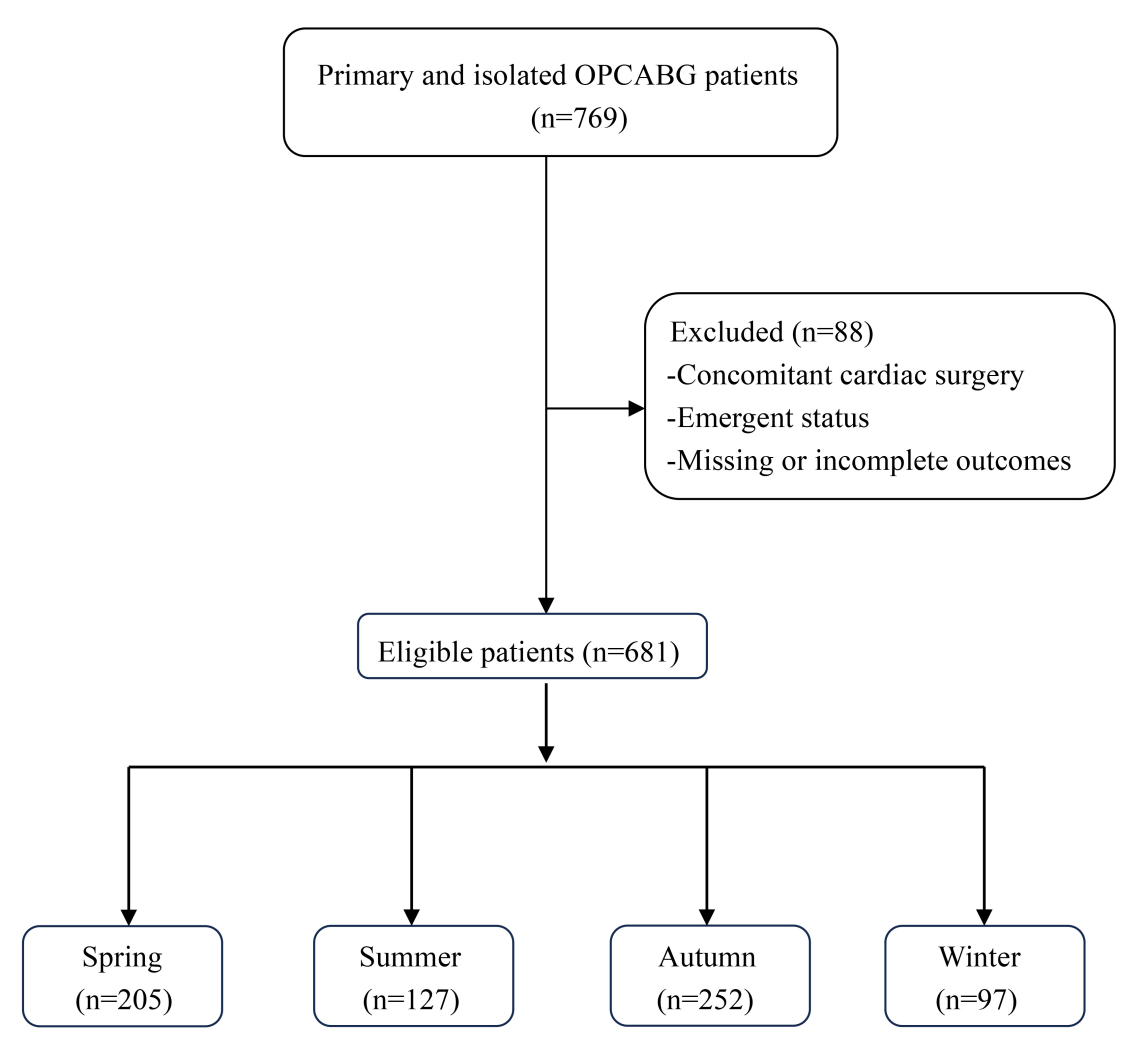
**Flowchart of the patient selection and categorization criteria**. OPCABG, off-pump coronary artery bypass graft.

The study included consecutive patients who underwent primary and isolated OPCABG at our hospital from January 2020 to December 2020. The inclusion criteria comprised adult patients (>18 years old) undergoing primary and isolated OPCABG. Patients with concomitant cardiac surgery, emergency status, and incomplete outcomes of interest were excluded. Out of the 769 patients who underwent OPCABG surgery during the study period, 681 were included based on the specified inclusion and exclusion criteria. These patients were divided into four groups according to the season of surgery. The classification of seasons followed established definitions: autumn (21st September to 20th December), winter (21st December to 21st March), spring (22nd March to 21st June), and summer (22nd June to 20th September) (Fig. 1).

### 2.2 Perioperative Management

Chronic cardiac medications were continued until the morning of surgery, with antiplatelet and anticoagulation medications discontinued preoperatively. No anesthetic premedication was administered. Standardized anesthetic protocols were employed, including induction with 0.2 mg/kg etomidate, 2–3 µg/kg sufentanil, and 0.15–0.2 mg/kg cis-atracurium, and maintenance with 100–200 mg/h propofol, 30–50 ug/h dexmedetomidine, 10–20 mg/h cis-atracurium, and 0.5%–2.5% sevoflurane. Surgical procedures involved a median sternotomy, with the left internal thoracic artery and/or great saphenous veins harvested for coronary grafts. A heparin dose of 200 U/kg was used to achieve an activated clotting time (ACT) of >300 seconds. Heparin was neutralized with protamine at a ratio of 0.5–0.8:1 at the end of revascularization. All patients received intraoperative autologous blood salvage by cell saver systems.

Blood products were transfused in adherence to the hospital’s transfusion protocol. An allogeneic red blood cell (RBC) transfusion was administered with hemoglobin (HB) <80 g/L. Fresh frozen plasma (FFP) infusion was indicated if diffuse bleeding with prothrombin time was 1.5 times longer than the baseline value. The transfusion trigger for a platelet concentrate (PC) transfusion included a platelet (PLT) count <50 × 10^9^/L or PLT dysfunction. Criteria for reoperation were postoperative chest tube drainage (CTD) volume >300 mL/within the first 2 hours or persistent drainage >200 mL/h for 4 hours. Extubation was performed under specified conditions, ensuring patient responsiveness, stable hemodynamics, and a CTD volume of less than 100 mL/h.

### 2.3 Endpoints

The primary outcome was the composite incidence of in-hospital mortality and morbidity, which included myocardial infarction (MI), low-cardiac output syndrome (LCOS), use of intra-aortic balloon pump (IABP), new-onset atrial fibrillation, ventricular fibrillation, new pacemaker placement, acute kidney injury (AKI), stroke, pneumonia, wound infection, reoperation, intensive care unit (ICU) readmission, and cardiac arrest. MI was defined as an increase in cardiac biomarkers (≥10 times the local laboratory upper limit of normal) in combination with one of the following: (1) new pathologic Q waves or new left bundle branch block; (2) angiographically documented new occlusion in the graft or native coronary artery; (3) imaging evidence of new loss of viable myocardium or new wall motion abnormality [10]. LCOS was defined by decreased heart pump function, resulting in reduced oxygen delivery (DO_2_), cardiac index (CI) ≤2.0 L/min/m^2^, systolic blood pressure <90 mmHg, and signs of tissue hypoperfusion [11]. AKI was defined as an increase in serum creatinine (≥0.3 mg/dL within 48 h or ≥1.5–1.9 times baseline level) or urine volume <0.5 mL/kg/h for 6 hours [12].

Secondary outcomes included CTD within 24 h, total CTD, chest drainage duration, mechanical ventilation duration, and postoperative LOS in the ICU and hospital.

### 2.4 Statistical Analysis

Continuous variables are expressed as the mean ± standard deviation if normally distributed or as the median and quartile range. Analysis of variance (ANOVA) or Wilcoxon–Mann–Whitney tests with the Bonferroni correction were used for comparisons. Dichotomous variables are presented as the frequency and percentage following analysis using chi-square or Fisher exact tests. All statistical analyses were performed using SPSS 27.0 (IBM SPSS Statistics 27.0, Chicago, IL, USA).

## 3. Results

Table 1 shows the baseline characteristics of the included OPCABG patients. There were no major differences in patient characteristics by season except for a disparity in preoperative usage of statins (*p* = 0.004).

**Table 1.** **Patient characteristics**.

			Spring (n = 205)	Summer (n = 127)	Autumn (n = 252)	Winter (n = 97)	*p*-value
Demographics							
	Age, y		64 (57, 69)	61 (55, 67)	64 (57, 70)	63 (54, 68)	0.142
	Male sex, n (%)		156 (76.1%)	104 (81.9%)	196 (77.8%)	77 (79.4%)	0.647
	BMI, kg/m^2^		25.4 (23.7, 27.4)	25.6 (23.5, 28.0)	25.9 (23.7, 27.7)	25.2 (23.2, 27.0)	0.292
Risk factor and comorbidities, n (%)							
	Smoking		97 (47.3%)	71 (55.9%)	123 (48.8%)	39 (40.2%)	0.134
	Drinking		10 (4.9%)	9 (7.1%)	25 (9.9%)	12 (12.5%)	0.088
	Hypertension		148 (72.2%)	82 (64.6%)	169 (67.1%)	62 (63.9%)	0.370
	Diabetes mellitus		91 (44.4%)	58 (45.7%)	99 (39.3%)	40 (41.2%)	0.585
	Hyperlipidemia		179 (87.3%)	98 (77.2%)	208 (82.5%)	75 (77.3%)	0.058
	COPD		3 (1.5%)	5 (3.9%)	3 (1.2%)	1 (1.0%)	0.299
	Atrial fibrillation		6 (2.9%)	8 (6.3%)	10 (4.0%)	3 (3.1%)	0.459
	Heart block		2 (1.0%)	1 (0.8%)	3 (1.2%)	2 (2.1%)	0.849
	Previous MI						
		≤6 m	4 (2.0%)	7 (5.5%)	11 (4.4%)	4 (4.1%)	0.316
		>6 m	25 (12.2%)	23 (18.1%)	47 (18.7%)	17 (17.5%)	0.270
	PCI		7 (3.4%)	6 (4.7%)	7 (2.8%)	7 (7.2%)	0.257
	Heart failure		3 (1.5%)	2 (1.6%)	2 (0.8%)	2 (2.1%)	0.702
	Renal failure		5 (2.4%)	3 (2.4%)	4 (1.6%)	3 (3.1%)	0.739
	Stroke		34 (16.6%)	17 (13.4%)	29 (11.5%)	8 (8.2%)	0.189
	Peripheral vascular diseases		15 (7.3%)	9 (7.1%)	8 (3.2%)	7 (7.2%)	0.186
Preoperative medications, n (%)							
	β-blockers		187 (91.2%)	118 (92.9%)	232 (92.1%)	87 (89.7%)	0.835
	Nitrates		189 (92.2%)	118 (92.9%)	241 (95.6%)	91 (93.8%)	0.466
	ACEI		27 (13.2%)	16 (12.6%)	49 (19.4%)	20 (20.6%)	0.120
	CCB		82 (40.0%)	47 (37.0%)	78 (31.0%)	36 (37.1%)	0.231
	Insulin		24 (11.7%)	16 (12.6%)	40 (15.9%)	20 (20.6%)	0.179
	Hypoglycemic agent		63 (30.7%)	39 (30.7%)	80 (31.7%)	27 (27.8%)	0.919
	Statins		188 (91.7%)	111 (87.4%)	230 (91.3%)	77 (79.4%)	0.006
	Aspirin		50 (24.4%)	32 (25.2%)	54 (21.4%)	18 (18.6%)	0.577
	Ticagrelor		2 (1.0%)	5 (3.9%)	6 (2.4%)	2 (3.7%)	0.225
	Clopidogrel		34 (16.6%)	24 (18.9%)	46 (18.3%)	7 (7.2%)	0.063
Operative parameters							
	Surgery duration, min		211 (175, 245)	210 (180, 255)	205 (175, 242)	207 (176, 240)	0.603
	Anesthesia duration, min		260 (222, 299)	260 (225, 300)	255 (225, 291)	250 (220, 289)	0.428
	Graft number		3 (3, 4)	3 (3, 4)	3 (3, 4)	3 (3, 4)	0.516

ACEI, angiotensin-converting enzyme inhibitor; BMI, body mass index; CCB, calcium channel blocker; COPD, chronic obstructive pulmonary disease; MI, myocardial infarction; PCI, percutaneous coronary intervention. Data are presented as the number (percentage) or median (interquartile range).

As shown in Table 2, the primary outcome exhibited significant differences (*p* = 0.004), with winter and spring surgeries revealing higher composite incidence of mortality and morbidities compared to the summer (15.7%) and autumn (11.1%) surgeries (*p* < 0.05). Spring surgery was associated with the highest median CTD at 24 h after surgery (640 mL), whereas it was the lowest total TCD (730 mL) compared to the other seasons (*p* < 0.05). Chest drainage duration was longer in spring and summer than in autumn and winter (*p* < 0.05). While no significant difference was observed in mechanical ventilation duration and hospital stay among the four seasons, LOS in the ICU was longer in summer than in autumn (88 h *vs.* 51 h, *p* < 0.05).

**Table 2.** **Postoperative outcomes of patients undergoing OPCABG in different seasons**.

		Spring (n = 205)	Summer (n = 127)	Autumn (n = 252)	Winter (n = 97)	*p*-value
Mortality and morbidity, n (%)		37 (18.0%)	20 (15.7%)	28 (11.1%)	26 (26.8%)	0.004
Mortality, n (%)		1 (0.5%)	0	1 (0.4%)	1 (1.0%)	0.651
Any morbidity, n (%)		36 (17.6%)	20 (15.7%)	27 (10.7%)	25 (25.8%)	0.006
	MI	1 (0.5%)	0	0	0	0.630
	LCOS	5 (2.4%)	0	0	1 (1.0%)	0.017
	IABP	1 (0.5%)	0	6 (2.4%)	2 (2.1%)	0.142
	Atrial fibrillation	0	2 (1.6%)	4 (1.6%)	2 (2.1%)	0.139
	Ventricular fibrillation	1 (0.5%)	0	1 (0.4%)	1 (1.0%)	0.651
	New pacemaker	3 (1.5%)	0	0	1 (1.0%)	0.084
	AKI	8 (3.9%)	1 (0.8%)	5 (2%)	4 (4.1%)	0.228
	Stroke	3 (1.5%)	4 (3.1%)	0	2 (2.1%)	0.022
	Pneumonia	15 (7.3%)	15 (11.8%)	15 (6.0%)	19 (19.6%)	0.001
	Wound infection	0	0	2 (0.8%)	0	0.639
	Reoperation	1 (0.5%)	0	0	1 (1.0%)	0.194
	ICU readmission	2 (2.0%)	0	0	1 (1.0%)	0.042
	Cardiac arrest	1 (0.5%)	0	0	0	0.630
CTD within 24 h, mL		640 (415, 955)	520 (335, 675)	480 (360, 650)	500 (350, 610)	<0.001
Total CTD, mL		730 (500, 1100)	940 (668, 1305)	970 (690, 1380)	880 (740, 1190)	<0.001
Chest drainage duration, d		4 (4, 5)	4 (4, 5)	4 (3, 5)	3 (3, 4)	<0.001
Mechanical ventilation duration, h		16 (13, 18)	16 (14, 18)	16 (13, 18)	16 (12, 19)	0.687
LOS in the ICU, h		69 (45, 98)	88 (60, 116)	51 (24, 96)	68 (29, 100)	<0.001
Postoperative LOS in hospital, d		7 (7, 9)	8 (7, 10)	7 (7, 10)	8 (7, 10)	0.213

AKI, acute kidney injury; CTD, chest tube drainage; IABP, intra-aortic balloon pump; ICU, intensive care unit; LCOS, low cardiac output syndrome; LOS, length of stay; MI, myocardial infarction; OPCABG, off-pump coronary artery bypass graft. Data are presented as the number (percentage) or median (interquartile range).

## 4. Discussion

This study sought to investigate the influence of seasonality on outcomes following OPCAB in a specific center. While mortality rates were not significantly affected by the season, our findings underscore a noteworthy impact on postoperative complications and ICU length of stay.

Winter emerged as a potentially unfavorable season for OPCABG, with postoperative pulmonary infection being the most prevalent alongside severe complications. Prior research has highlighted increased risks of postoperative pneumonia, influenza, and infections during the winter season [13, 14]. The incidence of pneumonia after OPCABG showed a strong seasonal pattern, which might also apply to other cardiothoracic surgical procedures [15]. Moreover, influenza season was identified as an independent risk factor for developing acute respiratory distress syndrome (ARDS) after cardiac surgery (odds ratio, 1.85; 95% confidence interval, 1.06 to 3.23) [16].

The underlying physiological mechanism driving seasonal variations in OPCABG outcomes appears rooted in the impact of cold exposure on coronary artery disease. Cold exposure may induce inflammatory and coagulation responses, particularly affecting vulnerable individuals [17]. Several climatic factors, such as light, temperature, humidity, and atmospheric pressure, can profoundly affect neurobiology, hormonal physiology, and cardiovascular function [18]. Furthermore, it has been reported that C-reactive protein levels show a seasonal dependence, being higher in winter and spring [19]. These factors may collectively contribute to a higher risk of postoperative complications when performing OPCABG in winter.

Interestingly, our study revealed that patients who underwent OPCABG in the summer tended to have longer ICU stays, which may be attributed to heat exposure on the body. Heat exposure can cause physiological changes, such as increased heart rate, blood viscosity, and coagulation [20], leading to poor prognosis after OPCABG, which may also be mediated by other factors such as the occurrence of heart failure, conduction disturbances, and arrhythmias [21, 22]. In addition, a lower rate of OPCABG surgeries was performed in winter, which was inconsistent with the epidemiology of coronary heart disease. This discrepancy may reflect the influence of the traditional Chinese festival of Spring Festival, as patients might favor conservative treatment or coronary stenting over OPCABG surgery due to traditional cultural values or beliefs [23].

Notably, although difficult to interpret, the variations in postoperative bleeding observed in our study raise intriguing questions about potential seasonal factors influencing hemostasis and coagulation [24]. Weather-related factors, patient physiology variations, or other seasonal dynamics may contribute to this phenomenon [25]. Thus, clinicians and surgical teams should be attuned to these seasonal nuances, adjusting strategies for managing intraoperative bleeding during autumn to optimize patient outcomes [26].

Our study has some limitations. First, as a retrospective cohort study, the methodology contains some inherent biases. Unmeasured or uncontrollable confounders remained, such as admission variations or staff differences. Second, the single-center characteristic of the study limits the generalizability and applicability of these results. Third, our study only addressed in-hospital events and did not capture long-term outcomes. Lastly, the study only describes the association between the seasons and OPCABG outcomes, meaning an explanation of causal mechanisms cannot be established.

## 5. Conclusions

In conclusion, our study provides valuable insights into the nuanced impact of seasonality on OPCABG outcomes in patients with coronary heart disease. More large-scale, multicenter, prospective studies are warranted to examine the effects of seasonal variation on the clinical outcomes of this population.

## Availability of Data and Materials

The datasets used and/or analyzed during the current study are available from the corresponding author on reasonable request.
